# The Difference in Pharmacists’ Interventions across the Diverse Settings in a Children’s Hospital

**DOI:** 10.1371/journal.pone.0110168

**Published:** 2014-10-08

**Authors:** Hesty Utami Ramadaniati, Ya Ping Lee, Jeffery David Hughes

**Affiliations:** School of Pharmacy and Curtin Health Innovation Research Institute, Curtin University, Bentley, Western Australia, Australia; Nottingham University, United Kingdom

## Abstract

**Aims:**

This study aimed to document and compare the nature of clinical pharmacists’ interventions made in different practice settings within a children’s hospital.

**Methods:**

The primary investigator observed and documented all clinical interventions performed by clinical pharmacists for between 35–37 days on each of the five study wards from the three practice settings, namely general medical, general surgical and hematology-oncology. The rates, types and significance of the pharmacists’ interventions in the different settings were compared.

**Results:**

A total of 982 interventions were documented, related to the 16,700 medication orders reviewed on the five wards in the three practice settings over the duration of the study. Taking medication histories and/or patient counselling were the most common pharmacists’ interventions in the general settings; constituting more than half of all interventions. On the Hematology-Oncology Ward the pattern was different with drug therapy changes being the most common interventions (n = 73/195, 37.4% of all interventions). Active interventions (pharmacists’ activities leading to a change in drug therapy) constituted less than a quarter of all interventions on the general medical and surgical wards compared to nearly half on the specialty Hematology-Oncology Ward. The majority (n = 37/42, 88.1%) of a random sample of the active interventions reviewed were rated as clinically significant. Dose adjustment was the most frequent active interventions in the general settings, whilst drug addition constituted the most common active interventions on the Hematology-Oncology Ward. The degree of acceptance of pharmacists’ active interventions by prescribers was high (n = 223/244, 91.4%).

**Conclusions:**

The rate of pharmacists’ active interventions differed across different practice settings, being most frequent in the specialty hematology-oncology setting. The nature and type of the interventions documented in the hematology-oncology were also different compared to those in the general medical and surgical settings.

## Introduction

Over the past decades, the traditional roles of clinical pharmacists have expanded from compounding, dispensing and supplying medicines to active participation in clinical activities. This expanded role has increased the contribution of pharmacists, as part of health care team, to minimize drug-related problems and optimize patient outcomes [1]. Studies show that pharmacists are crucial in medication misadventure reporting and they play an important role in medication error prevention, especially when participating in patient rounds [2]. Many specialist clinical units such as neonatal critical care and oncology have relied on pharmacist participation during clinical rounds in order to resolve issues of adverse drug reactions, drug interactions and medication errors associated with complexity of medical conditions and medication regimens used in these units [3], [4]. Clinical pharmacy practices are prevalent in hospital settings in developed countries like Australia, the United Kingdom, and the United States; yet the nature and extent of the services provided appear to be highly variable [5]–[7].

The precise meaning of the term pharmacists’ intervention is still occasionally debated, but in Australia, there is generally broad acceptance that a clinical pharmacy intervention is ‘any action by a clinical pharmacist that directly results in a change in patient management or therapy’ [8]. This definition appropriately acknowledges that clinical pharmacists’ interventions only occur if the pharmacist is able to influence the behavior of the prescriber and other health care professionals such as nurses. Nevertheless, it should be noted that in addition to intervening to resolve/prevent actual/potential drug related problems, clinical pharmacists perform a range of other important functions that might not necessarily result in recommendations or direct changes in medication management but these functions could rightly be described as clinical pharmacy interventions [9]. For the purpose of this study, the term of pharmacist’s intervention refers to any action by a clinical pharmacist related to patient management or therapy and these can be classified as active and passive [8]. Passive interventions refer to care-centered activities not resulting in medication changes and active interventions are those activities leading to a change in drug therapy.

Previous studies have reported the contribution of clinical pharmacists to patient care in a variety of clinical settings in pediatrics [10]–[13]. In general, intervention data from those studies has been aggregated for the purpose of analysis rather than presented according to each clinical setting. Maat et al [10], did however reported that in a study of interventions associated with electronic prescription review, interventions were most frequently conducted in the specialty units of immunology/hematology and neurology compared to other units such as internal medicine. Similarly, Barber et al [14], in a study involving children and adults, reported higher rates of interventions in intensive care and pediatrics/specialty wards (e.g. hematology, oncology, AIDS, organ transplant) compared to other ward types. Therefore, this study was conducted to document and compare the nature of clinical pharmacists’ interventions made within different clinical settings in a children’s hospital.

## Methods

### Setting

This study was conducted in a 220-bed pediatric teaching hospital in Perth, Western Australia. Data were collected using prospective non-disguised observational approach from September 2011 to August 2012 from three practice settings, namely general medicine, general surgery and hematology-oncology. There were three wards under general medicine, namely the General Medical Ward for Infants, the General Medical Ward for Young Children, and the General Medical Ward for Adolescents. There was one ward assigned to general surgery and one to hematology-oncology. The general medical wards are the acute medical wards admitting patients under general pediatrics and a range of non-oncology medical specialties, whilst the general surgical ward admits patients under general surgery, ophthalmology and otolaryngology. The principal researcher observed and documented all interventions undertaken by clinical pharmacists during their pharmacy rounds.

### Pharmacists’ Interventions

During the data collection period, the primary investigator observed and documented all clinical interventions performed by the clinical pharmacists for between 35–37 days on each of the five study wards from the three practice settings. Observation on non-consecutive days was undertaken to avoid possible observation fatigue of the pharmacists. During observation, the data collected included the patient’s demographics, date of admission, diagnosis on admission, medical history, medication history, adverse drug reaction history, current medications, discharged date, the description and the type of intervention, the medication(s) involved, the cause of the intervention, the trigger of the intervention, the intervened health care personnel, the degree of acceptance of the intervention and the amount of time required to do pharmacy rounds. The diagnosis on admission was classified using the International Statistical Classification of Diseases and Related Health Problems 10^th^ Revision [15] for general medical and general surgical wards, and the International Classification of Childhood Cancer 3^rd^ Edition/ICCC-3 with slight modifications for diagnosis on Hematology-Oncology Ward [16]. The medications involved in the interventions were categorized using the Australian Medicines Handbook (AMH) 2014 drug classes [17]. The types of interventions were categorized into major types with further sub-categorization as described by Condren et al with slight modifications [18]. The rates of interventions were defined as the number of interventions per 100 medication orders reviewed. In addition, the interventions were divided into active and passive interventions as defined previously. The clinical significance of randomly selected sample of pharmacists’ active interventions (approximately 16% of active interventions) was assessed by panel consisting of two of the investigators and two independent hospital pharmacists. The consensus among the panel members was used for the final rating.

### Ethics Statement

This study was approved by the Princess Margaret Hospital Institutional Review Board and Curtin University Human Ethics Committee (No: PH-14-11). Written informed consent has been obtained from the observed pharmacists prior to the commencement of the study.

### Data Analysis

Demographic variables and pharmacists’ intervention data were summarized using descriptive statistics (mean ± standard deviation or median and range for variables measured on a continuous scale, and frequencies and percentages for categorical variables). Several pharmacists’ intervention related parameters among the three practice settings were compared using Kruskal-Wallis analysis. Data were analyzed using the SPSS version 19.0 (Chicago, IL, USA). The rates of pharmacists’ total interventions and active interventions in the three settings were compared using Poisson regression analysis using SAS version 9.2 (SAS Institute Inc., Cary, NC). Poisson regression analysis was also performed to determine the influence of the duration of pharmacy ward rounds on pharmacists’ intervention rates.

## Results

During the observation period of the study, 2891 patients were reviewed by the clinical pharmacists in the three practice settings. The basic demographic data of these patients are detailed in Table 1. A total of 982 interventions were observed and documented, which arose from 16,700 medication order reviews. The breakdown of the pharmacists’ intervention data from each clinical setting is summarized in Table 2 and the types of interventions performed by the clinical pharmacists on the study wards are outlined in Table 3. In terms of triggers leading to interventions, the most frequent trigger was medication chart review, with this monitoring activity being responsible for the majority of interventions (79.3%) across all clinical settings.

**Table 1:** Demographic Data of Patients in Three Clinical Settings.

Parameters	General Medicine	General Surgery	Hematology-Oncology
Duration of study (days)	105	37	35
No. of patients	1936	514	441
Gender (%)			
Male	939 (48.5)	311 (60.5)	279 (63.3)
Age* (years)	8.04 (0.02–19.00)	6.17 (0.06–17.00)	6.83 (0.35–17.00)
Length of stay* (days)	9.00 (1–95)	5.00 (1–71)	7.00 (1–82)
Types of medications*			
Oral	3.00 (0–22)	3.00 (0–10)	4.00 (0–21)
Non-oral	1.00 (0–30)	2.00 (0–13)	3.00 (0–14)

*Median (Range).

**Table 2:** The Pharmacists’ Interventions Documented in Three Clinical Settings.

Parameters	General Medicine	General Surgery	Hematology-Oncology	P-value
Duration of pharmacy round (minutes)#	50.5±23.3	50.7±19.0	41.7±20.1	0.042*
	(46.0–55.0)	(44.4–57.0)	(34.8–48.6)	
No. of pharmacists’ interventions	516	271	195	-
Total number of medication orders reviewed	10494	2700	3506	-
No. of medication orders reviewed/day#	99.9±40.6	72.9±21.1	100.2±33.0	0.001*
	(92.1–107.8)	(65.9–80.0)	(88.8–111.5)	
Rate of Pharmacists’ interventions per day#	4.9±3.8	7.4±0.8	5.6±0.6	0.014*
	(4.2–5.7)	(5.9–9.0)	(4.4–6.8)	
Rate of Pharmacists’ interventionsper 100 medication orders reviewed#	5.7±5.1	10.5±1.1	5.6±0.6	<0.001§
	(4.7–6.7)	(8.3–12.7)	(4.5–6.8)	

# Denotes: Mean ± Standard Deviation (95% Confidence Interval for Mean).

*Statistical analysis using Kruskal-Wallis test.

§ Statistical analysis using Poisson regression.

**Table 3:** Types of Pharmacists’ Interventions Documented in the Three Clinical Settings.

Intervention Categories [18]	Number of Pharmacists’ Interventions (%)		
	General Medicine	General Surgery	Hematology-Oncology
	(n = 516)	(n = 271)	(n = 195)
Medication history and/or patient counselling	307 (59.5)	146 (53.9)	48 (24.6)
Drug therapy changes	65 (12.6)	50 (18.5)	73 (37.4)
Provision of drug information to other providers	57 (11.0)	27 (10.0)	52 (26.7)
Clarification of medication orders	52 (10.1)	35 (12.9)	3 (1.5)
Prevented adverse drug event (ADE) or medication error (ME)	23 (4.5)	8 (3.0)	16 (8.2)
Other	6 (1.2)	3 (1.1)	2 (1.0)
Occurrence of ADE or ME)	6 (1.2)	0	1 (0.5)
Drug interaction	0	1 (0.4)	0
Laboratory monitoring	0	1 (0.4)	0

As can be seen from the data in Table 2, the clinical pharmacists made approximately 5–7 interventions per day. The rates of intervention ranged from 5.63 to 10.48 interventions per 100 medication orders reviewed across the different settings. The Poisson regression model showed that the rates of intervention were significantly different across the three practice settings (p<0.001). The rate of interventions appeared to be the highest in general surgery, followed by general medicine and hematology-oncology. It was observed that there was no significant difference in the rates of interventions between general medicine and hematology-oncology (p = 0.202), but the rate of intervention in general surgical setting was significantly higher (P<0.001) than that of the two other settings. On average, the pharmacists spent approximately 49 minutes per ward round. The more time spent on the ward rounds significantly increased the rate of interventions across all clinical settings (p<0.001).

As can be seen from Table 3, taking medication histories and/or patient counselling were the most common interventions performed by the clinical pharmacists in the general medical and surgical settings, with these activities constituting more than half of all interventions. In contrast, the specialty unit, namely hematology-oncology, had a different pattern of interventions; here drug therapy changes were the most common interventions, representing around 37% of all interventions.

### Active Pharmacists’ Interventions, Degree of Acceptance, and Implicated Medications

Hematology-oncology had the highest rate of active interventions (2.43/100 medication orders reviewed), followed by general surgery (2.34) and general medicine (0.93). The rate of active interventions in hematology-oncology was significantly higher compared to general medicine (p<0.001) but not with general surgery (p = 0.331). Meanwhile, the rates of active interventions were not significantly influenced by the time spent on the ward (p = 0.187). With regards to the clinical significance of active interventions, the majority (n = 37/42, 88.1%) were rated as being clinically significant. The significance of the interventions ranged from not significant (12.8%), minor (46.2%), moderate (23.1%) and major (17.9%); with no intervention thought to be life-saving. Active interventions constituted less than a quarter of all interventions on the general medical and surgical wards, compared to 46.2% (p<0.001) on the Hematology-Oncology Ward. Table 4 shows the distribution of the 244 active interventions by type. For all active interventions, the degree of acceptance was high at 91.4% (i.e. 223/244). “Adjusting the dose” was the most frequent type of active intervention on both the general medical and surgical wards. A slightly different trend was found on the Hematology-Oncology Ward where the recommendations to prescribe regular medications constituted the most common active interventions (40%) followed by dose adjustment (26.7%). With respect to the classes of medications implicated in active interventions, anti-infectives were the drugs most often associated with active interventions (n = 100), followed by analgesics (n = 46) and drugs for the gastrointestinal system (n = 36), respectively.

**Table 4:** Types of Active Interventions in the Three Clinical Settings.

Types of interventions	Number of Active Interventions		
	General Medicine	General Surgery	Hematology-Oncology
	(n = 95)	(n = 59)	(n = 90)
Wrong/missing dose	36	21	24
Wrong/missing dosage interval/frequency	18	7	6
Drug added	10	8	36
Drug deleted	15	6	5
Antibiotic change	2	2	0
Wrong/missing therapy duration	1	0	6
Wrong/missing dosage form or strength	6	4	1
Regular to if required/if required to regular	1	6	3
Wrong/missing route	2	0	0
Scheduling error	1	0	3
Non formulary to formulary	0	3	0
MAR§ error	1	0	4
Other#	2	2	2

*Rates of active intervention per 100 medication orders, p value <0.001 based on statistical analysis using Poisson regression.

§ Denotes medication administration record.

# Other category includes wrong drug, intravenous to per oral change, wrong patient, illegible order, drug interaction.

## Discussion

The prevalence of pharmacists’ interventions per 100 medication orders reviewed in this study ranged from 5.66 in general medicine, 10.48 in general surgery, and 5.63 in hematology-oncology The rate of intervention in the general surgical setting in our study was the highest compared to the other two settings (p<0.05), although the surgical setting is often considered less complex than general medical, intensive care or specialty areas [19]. In comparison to the rates recorded in general medical settings in our study, a higher active intervention rate (2.4/100 medication orders) was reported from a study assessing the impact of interventions performed by clinical pharmacists in reducing prescribing errors in children hospitalized in a maternity and children’s hospital in Spain [11]. However, the rate of active interventions found on the Hematology-Oncology Ward in our study was similar to that of the Spanish study. Unfortunately, the Spanish study did not provide further information regarding the nature of patients’ medical conditions. Presumably, active intervention rates are influenced by the complexity of patients’ medication regimens.[10] This may account for our finding where a higher incidence of active interventions was observed in the hematology-oncology setting compared to other settings where patients may have less complex conditions requiring less medication.

Aside from the number of medications prescribed, other factors need to be taken into account as the predictors for pharmacists’ intervention rate. Consistent with our study, a large pharmacists’ interventions study in the UK, which included pediatric and adult patients, found the time spent during ward rounds significantly predicted the rates of the interventions [14]. In relation to the clinical significance of active interventions, other studies involving pediatric patients have had similar findings with around three-quarters of the interventions having a positive impact on patient care [11], [20], [21].

With respect to the pattern of pharmacists’ interventions, activities related to taking medication histories and/or patient counselling were the most frequent interventions in general settings, whilst drug therapy changes were responsible for the most frequent interventions in hematology-oncology setting. With respect to intervention category, the subcategory of taking medication histories and medication reconciliation activities constituted the most common interventions performed by clinical pharmacists in the general medical and surgical settings throughout the study. This is not surprising given taking medication histories and medication reconciliation are the initial steps in reviewing patients and assessing the appropriateness of their medications orders [22]–[24]. However, these activities occurred less frequently in the hematology-oncology unit. The lower proportion might be explained by the availability, completeness and the ease of access to patients’ medical and medication histories in this specialty setting. In addition, as the hematology and oncology patients were admitted regularly to hospital for treatment and monitoring, the health care providers including pharmacists were familiar with the patients and their treatment protocols so the pharmacists often looked at the patients’ records instead of interviewing the patients and/or the parents.

There are limited published studies on pharmacists’ interventions in a range of different clinical settings in pediatrics compared to adult patients. Condren et al [23] conducted a self-reported intervention study in a pediatric patient population including general and intensive care inpatients and ambulatory patients. The study reported 1.15 interventions per patient and the most frequent intervention categories were drug therapy change, taking medication history and/or patient counselling, and providing drug information to other health care providers [18]. In addition, the categories of common active interventions found by Condren et al were consistent with the pattern of active interventions in the hematology-oncology setting in our study [18].

In a 4-week study in 16 pediatric wards (9 specialist and 7 general wards) in the United Kingdom, interventions to resolve improper dosing, incomplete prescriptions, and wrong frequency were documented as the most common interventions performed by pharmacists [25]. Dosing issues were also identified as the main problem requiring pharmacists’ intervention in hospitalized children in a Spanish study [11]. Likewise, a retrospective analysis of 4 years of self-reported pharmacists’ interventions conducted by Chan and colleagues involving pediatric patients in general and specialty areas including hematology-oncology also detected dosing issues as the major source of problems [13]. These studies incorporating general and specialty settings [11], [13], [18], [25] have consistently shown that dosing-associated issues are the main problem in pediatric patients. However those studies did not provide further information regarding the breakdown of interventions for each clinical setting so the pattern and rate of interventions among the different settings, in particular general versus specialty settings, in the child patient populations cannot be compared and analyzed comprehensively. Nonetheless, a Dutch study uncovered that the practice settings did influence the intervention rate with interventions more common in immunology/hematology and neurology compared to internal medicine [10]. Further, Kaushal et al who conducted a study to assess the rate of serious medication errors before and after the introduction of unit-based clinical pharmacists in three units (intensive care unit/ICU, general surgery and general medicine) in a US pediatric hospital, reported that clinical pharmacists’ interventions substantially decreased the rate of medication errors in the ICU whilst there was no rate reduction in the general settings. The investigators pointed out that the setting influenced the rates of medication error-intercepting pharmacists’ interventions, in particular between the general and non-general settings such as ICU [12].

In addition, the acceptance rate of the pharmacists’ active recommendations was high across all settings in our study. This rate of acceptance is similar to those found in other pediatric studies [11], [21], [26]. This strengthen the established evidence supporting the confidence of other health care providers have in the significant contribution of pharmacists to improve the quality of patient care in pediatric settings [12], [18], [27], [28].

To the best of our knowledge, this is the first study that compared the pharmacists’ interventions in a range of different settings in pediatrics in terms of frequency, type, degree of acceptance, clinical significance and medications implicated. It is worthwhile noting that the majority of earlier studies on pharmacists’ interventions were conducted using a self-reporting approach by the intervening pharmacists. The prospective observational approach used in this study allowed the observer to obtain the actual number of interventions performed by ward pharmacists to overcome the issues with self-reporting [29], [30]. Nonetheless, a number of limitations need to be acknowledged in this study. This study has been conducted in one pediatric hospital which diminishes the ability to generalize the findings. Further, the data has been collected on non-consecutive days to avoid pharmacist observation fatigue which may influence the pattern of the interventions. Another limitation is the difficulty in drawing accurate comparisons with other intervention studies due to considerable variations in settings, design, duration, size, methodology and definition of intervention.

In conclusion, the rate and nature of pharmacists’ interventions appears to be influenced by the clinical setting. Specialty units, in this case hematology-oncology, had a higher active intervention rate as a result of more interventions related to drug therapy changes compared to general medical and surgical units. The findings of this study highlight the significance of pharmacists’ interventions in optimizing patient care in a range of pediatric settings.
